# First-Principles Investigations of Low-Dimensional Nanomaterials (2nd Edition)

**DOI:** 10.3390/nano16211408

**Published:** 2026-11-02

**Authors:** Fengyu Li, Jingxiang Zhao

**Affiliations:** 1 School of Physical Science and Technology, Inner Mongolia University, Hohhot 010021, China; 2 Key Laboratory of Photonic and Electronic Bandgap Materials, Ministry of Education, College of Chemistry and Chemical Engineering, Harbin Normal University, Harbin 150025, China; zhaojingxiang@hrbnu.edu.cn

## Editorial for the Special Issue of *Nanomaterials*

Low-dimensional nanomaterials, including zero-dimensional (0D) nanoclusters, one-dimensional (1D) nanoribbons and nanotubes, and two-dimensional (2D) nanosheets, exhibit distinctive physical and chemical properties with potential applications in nanoelectronics, quantum technologies, catalysis, and energy conversion. Quantum confinement, reduced atomic coordination, edge effects, and high surface-to-volume ratios give rise to behavior that differs from that of their bulk counterparts. These properties can be tuned through structural design, doping, defect engineering, interfacial interactions, and external stimuli. Establishing the microscopic mechanisms that connect the atomic structure to material properties is therefore essential for designing functional nanomaterials.

First-principles calculations, particularly those based on density functional theory (DFT), provide an atomic-scale framework for predicting structures, characterizing properties, and explaining the mechanisms underlying material behavior. Their integration with nonequilibrium Green’s function (NEGF) methods, ab initio molecular dynamics, high-throughput workflows, and machine learning extends this framework to electronic transport, finite-temperature dynamics, and the exploration of complex structural spaces. Together, these approaches connect fundamental understanding with the computational design of materials and devices.

Following the successful first edition, this Special Issue, entitled “First-Principles Investigations of Low-Dimensional Nanomaterials (2nd Edition)”, brings together seven original research articles on electrocatalysis, electronic transport, resistive switching, topological materials, photocatalysis, and machine-learning-assisted structure prediction. The collection examines how local bonding, defects, interfaces, and structural topology govern material properties. Although its primary focus is on low-dimensional systems, it also includes bulk materials and three-dimensional frameworks that illustrate complementary strategies for atomic-scale materials design.

Catalysis and energy conversion represent important applications of low-dimensional nanomaterials, whose exposed active sites and tunable electronic structures offer opportunities for developing efficient and sustainable catalysts. Guo et al. [1] systematically investigated several 2D silicon carbide monolayers as metal-free electrocatalysts for nitrate reduction to ammonia using DFT calculations. Among the investigated candidates, the Si_3_C monolayer exhibited particularly promising catalytic performance, with a predicted limiting potential of −0.43 V. This remarkable activity originated from the cooperative interaction among three neighboring Si active sites, which facilitated nitrate adsorption, activation, and subsequent hydrogenation, while suppressing the competing hydrogen evolution reaction. This study demonstrates that appropriately arranged nonmetallic active sites can provide catalytic functionalities traditionally associated with transition metals, offering a promising strategy for designing sustainable electrocatalysts.

Another contribution addressing energy-related applications was reported by Zhao et al. [2], who investigated the effects of Cd, Sn, and Hf doping on the electronic and optical properties of K(Ta_0.5_Nb_0.5_)O_3_ ferroelectric materials. Their first-principles calculations indicated that metal substitution induces lattice distortion, narrows the band gap, and shifts optical absorption toward lower photon energies. Changes in the dielectric response and carrier effective masses also suggest possible routes to improved photogenerated charge separation and photocatalytic performance. Although the system is a bulk ferroelectric, the study illustrates how the chemical modification of local atomic environments can tune properties relevant to energy conversion.

Quantum transport and nanoelectronic devices constitute another central theme of this Special Issue. As conventional semiconductor devices approach their miniaturization limits, atomically thin materials provide promising platforms for developing energy-efficient electronic components. However, their practical performance depends critically on electrode contacts, interfacial interactions, and atomic defects.

Zou et al. [3] investigated the interfacial properties of anisotropic monolayer SiAs transistors with graphene, V_2_CO_2_, Au, Ag, and Cu electrodes by combining first-principles electronic-structure calculations with quantum transport simulations, demonstrating that the contact characteristics depend strongly on the electrode material. In particular, monolayer SiAs forms Schottky contacts with several conventional electrodes, whereas V_2_CO_2_ was predicted to provide an ideal ohmic contact. These findings highlight the importance of interface engineering and electrode selection in overcoming contact-related limitations in 2D semiconductor devices.

Defect engineering offers another effective route for controlling electronic transport. He et al. [4] explored the resistive switching mechanism of Ta/h-BN/Ta atomristors using DFT combined with NEGF calculations. They demonstrated that the interplay between boron vacancies, Ta adsorption, and interfacial electronic states can govern the transition between high- and low-resistance states. In particular, Ta atoms interacting with boron vacancies modify the interfacial tunneling barriers and introduce electronic states within the band gap, thereby enhancing electron transmission. Their work provides microscopic insights into resistive switching at the atomic limit and offers theoretical guidance for developing ultrathin nonvolatile memories and neuromorphic computing devices.

Along similar lines, Song et al. [5] investigated the transport properties of arsenene nanoribbon tunnel field-effect transistors (TFETs) incorporating hydrogen defects and gate-all-around architectures. Their calculations showed that appropriately positioned H defects can substantially enhance ON-state currents and reduce the subthreshold swing below 60 mV/decade. Furthermore, combining defect engineering with gate-all-around electrostatic control improved device performance, achieving a predicted subthreshold swing as low as 35 mV/decade. These findings demonstrate that the deliberate introduction of atomic defects, when combined with optimized device architectures, can provide an effective strategy for developing low-power nanoelectronic devices.

Novel quantum materials and computational materials discovery provide further opportunities for first-principles investigations. Liu et al. [6] proposed a novel nanoporous germanium allotrope, I229-Ge_48_, constructed through bottom-up cluster assembly. Their calculations revealed unconventional topological nodal-line features, pronounced mechanical anisotropy, and strain-tunable electronic and optical properties. Notably, the compressive strain was predicted to induce a transition from a semimetallic to a semiconducting state, whereas the characteristic topological features remained under tensile strain. The study illustrates how cluster-based structural design and strain engineering can be employed to explore unconventional quantum states and multifunctional materials. Although I229-Ge_48_ forms a three-dimensional framework, its construction from nanoscale building blocks highlights the broader potential of bottom-up materials design.

Meanwhile, the integration of first-principles calculations with machine learning is introducing new possibilities for efficiently exploring complex configurational spaces. Sai et al. [7] developed a graph neural network model to predict the energetically favorable structures of boron- and nitrogen-substituted C_60_ fullerenes. By incorporating atomic connectivity and ring topology into a model trained on DFT data, they achieved accurate and efficient energy predictions for large numbers of structural isomers. The approach enabled the identification of previously unreported lower-energy configurations of heterofullerenes, demonstrating the potential of combining first-principles calculations with artificial intelligence to accelerate global structural searches and materials discovery.

Despite their diverse research objectives, these seven contributions share a common scientific foundation: the physical and chemical functionalities of materials are ultimately governed by their atomic configurations, chemical bonding, electronic structures, and interactions with their environments. The studies on Si_3_C electrocatalysts and doped ferroelectrics demonstrate how local chemical environments regulate catalytic and optical properties [1,2]. The investigations of SiAs transistors, h-BN atomristors, and arsenene nanoribbon TFETs reveal the crucial roles of interfaces and defects in controlling quantum transport [3,4,5]. Meanwhile, the exploration of cluster-assembled germanium and machine-learning-assisted fullerene structures illustrates the power of structural design and computational prediction in discovering unconventional materials and properties [6,7].

Looking forward, several challenges and opportunities deserve particular attention. First, more accurate descriptions of electronic correlations, excited states, and finite-temperature effects are needed to improve the reliability of theoretical predictions. Second, realistic environmental conditions, including substrates, interfaces, defects, strain, and external electric fields, should be incorporated into computational models to establish closer connections with experiments. Third, integrating first-principles calculations with machine learning, automated high-throughput workflows, and experimental feedback will further accelerate the discovery of functional nanomaterials. Finally, developing a deeper understanding of the relationships among atomic structures, electronic interactions, and device-level performance will be essential for translating computational predictions into practical applications.

In summary, the contributions collected in this Special Issue highlight the continuing importance and expanding capabilities of first-principles investigations in nanomaterials research. We hope that these studies will stimulate further interdisciplinary efforts in condensed matter physics, chemistry, materials science, and computational science and inspire new strategies for the rational design of low-dimensional nanomaterials and emerging functional systems.
